# Spontaneous haemorrhage of chronic varicose ulceration causing ischaemic colitis

**DOI:** 10.1093/jscr/rjab617

**Published:** 2022-02-02

**Authors:** Jonathon Holt

**Affiliations:** General Surgery, Albury-Wodonga Health, Albury, New South Wales, Australia

**Keywords:** venous insufficiency, chronic wound, spontaneous haemorrhage

## Abstract

Venous ulcers are common and account for the majority of lower limb chronic wounds. Complications may be serious in nature and although haemorrhage is rare, it may be rapidly fatal. The case of a female patient who presented with a spontaneous varicose ulcer haemorrhage causing hypovolaemic ischaemic colitis is described. This case exemplifies the need to identify and manage the development of venous insufficiency before progression to chronic wounds or other disease burdens which may be fatal or life-altering.

## INTRODUCTION

Serious spontaneous haemorrhage from a varicosity underlying a venous ulcer is a relatively rare phenomenon despite venous ulcers being very common and accounting for most lower limb chronic wounds. The condition carries significant morbidity and economic burden, a reflection of the limited success in treatment. Complications of venous ulceration may be serious in nature and although haemorrhage is rare, it may be rapidly fatal. The case of a female patient who presented with a spontaneous varicose ulcer haemorrhage causing hypovolaemic ischaemic colitis is described. Similar cases in the literature were reviewed and the clinico-pathological correlates were compared. This case exemplifies the need to identify and manage the development of venous insufficiency before progression to chronic wounds or other disease burdens which may be fatal or life-altering.

## CASE REPORT

A 75-year-old-woman was brought by ambulance to a rural urgent care centre with sudden onset of venous ulcer haemorrhage. The patient had a history of a non-healing ulcer above the right malleolus, with secondary to venous insufficiency. Duplex ultrasound prior to presentation had demonstrated an incompetent great saphenous vein which communicated with local varicosities and the ulcer. A prior wound biopsy was consistent with stasis dermatitis.

The primary survey confirmed a patent airway. Breathing was laboured with a respiratory rate of 30 breaths per minute. Oxygen saturation via pulse oximetry was recorded at 91% supported with 4 l/min 0_2_ via Hudson mask. The patient was peripherally cold, tachycardic at 100 beats/min and had an unrecordable blood pressure after an initial recording of 85/60 mmHg en route in the ambulance. A compression bandage soaked in blood was noted on the right leg. The GCS was 14 (E4V4M6) and the temperature was 35.1°C.

Initial point of care investigations were obtained with an iSTAT analyser and were as follows: haemoglobin: 105 g/l, haematocrit: 0.31 l/l, sodium: 140 mmol/l, potassium: 3.3 mmol/l, pH: 7.282, pC0_2_: 34.9 mmHg, HC0_3_: 16.9 mmol/l, lactate: 10.9 mmol/l and BSL: 18. An electrocardiogram demonstrated sinus rhythm.

The patient was resuscitated with fluid, and the bleeding ulcer was managed with a compressive bandage. After initial stabilization, a secondary survey was carried out. The respiratory rate had dropped to 20, Sa02 to 100% on RA and BP to 115/80 mmHg with a heart rate (HR) of 80 bpm. The respiratory and cardiovascular examinations were normal. The gastrointestinal exam revealed epigastric pain to deep palpation. The patient was orientated to time, person and place and had no focal neurological deficit.

**Figure 1:**
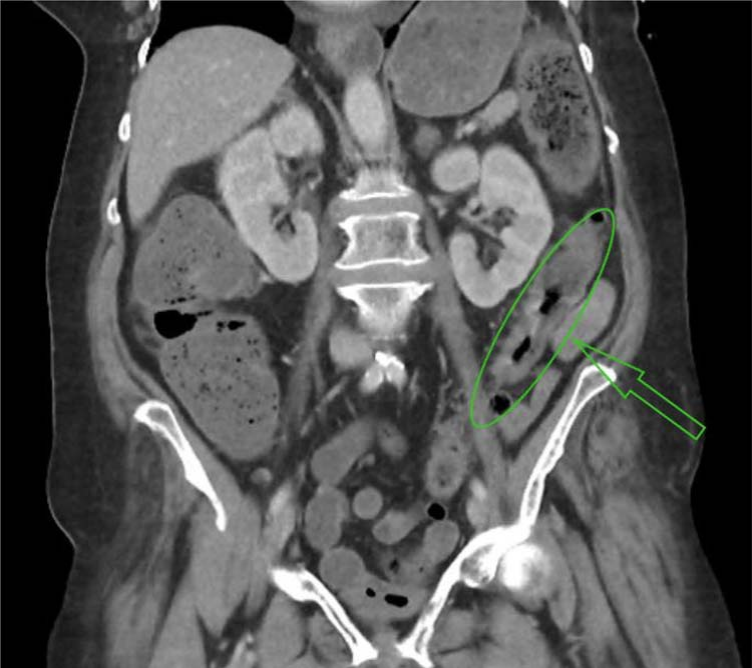
Mural thickening of sigmoid colon.

In the context of abdominal pain and a high lactate secondary to hypovolaemic shock, the patient was transferred to the nearest hospital with on-call surgical services. On arrival, a computed tomography of the abdomen and pelvis demonstrated mural thickening of the descending and proximal sigmoid colon, reported as possibly subtle ischaemic or inflammatory change (Fig. 1). The patient was taken to theatre for a laparotomy and was found to have extensive ischaemic large bowel requiring total colectomy and end ileostomy. Post-operatively, the patient was transferred to the ICU, requiring high levels of inotropic support; however, eventual step-down care was achieved and the patient was discharged home.

## DISCUSSION

Venous ulcers are the most common cause of chronic lower limb wounds [1]. The prevalence increases with age, and it is estimated that up to 2% of the general population will be affected [2]. The pathogenesis is principally related to chronic venous insufficiency, the cause of which is multifaceted, but is classically a result of venous hypertension due to chronic valvular reflux [3]. Whether the incompetence originates because of primary valvular failure, or the valves fail because of a process causing dilation of the vein is unclear [4, 5]. Venous obstruction, thrombophlebitis, congenital valve absence, vein wall fascial weakness and increased distensibility due to circulating oestrogens have all been proposed as contributing pathophysiological mechanisms [6]. The manifestations of chronic venous insufficiency include varicose veins, oedema, varicose eczema, lipodermatosclerosis, acute ulceration and chronic ulceration. Risk factors for chronic venous insufficiency include advancing age, family history, smoking, long standing hours, obesity, pregnancy and DVT.

Two forms of ulcer related to chronic venous insufficiency have been discussed in comparative case reports, both of which may haemorrhage spontaneously. These are the acute perforating type and the chronic ulcerative type, which differ clinically and histologically [6]. The acute perforating ulcer occurs early in the process of chronic venous insufficiency and is the result of an acute ulceration of an underlying varicosity through the skin [6]. These ulcers are usually <5 mm in diameter and are usually only associated with mild skin changes of chronic venous insufficiency demonstrating slight fibrosis and trophic changes [7]. Small scars present on the lower limbs in patients who have had spontaneous haemorrhage have also been reported, suggesting previous acute ulcerative processes that had healed quickly without concern [8]. Jelev and Alexandrov propose that local vein thrombosis, predominantly on the skin side, leads to the thickening and incorporation of the thrombus, followed by epidermal hyperplasia and inflammation, allowing the formation of a weak point which may lead to outward rupture and haemorrhage.

The chronic ulcerative process is characterized by a large and deep ulcer with significant surrounding skin changes, including fibrosis, pigmentation and stasis dermatitis [8]. These ulcers appear later in the course of chronic venous insufficiency and heal poorly. They may erode into underlying varicosities such as the long saphenous or short saphenous veins which lie deep to the superficial fascia [8, 9].

A review of fatal cases of spontaneous venous ulcer haemorrhage by Doberentz *et al*. identified that patients tended to be elderly and socially isolated. Concomitant factors, such as dementia, polypharmacy, anticoagulation, alcohol and drugs, also predispose to fatal haemorrhage. Evans *et al*. noted that simple treatment of non-fatal haemorrhage with compression bandaging was likely to be insufficient and cited a number of cases where fatal haemorrhage had been preceded by non-fatal haemorrhage.

## CONCLUSION

Treating practitioners should be mindful of the burden of disease presented by chronic venous insufficiency. Varicosities have the potential to haemorrhage which can be fatal. Early detection and primary management is key in preventing sequelae of disease.

## CONFLICT OF INTEREST STATEMENT

None declared.

## FUNDING

None.
